# Women want Pre-Exposure Prophylaxis but are Advised Against it by Their HIV-positive Counterparts

**DOI:** 10.4172/2155-6113.1000522

**Published:** 2015-11

**Authors:** Lakshmi Goparaju, Laure S Experton, Nathan C Praschan, Lari Warren-Jeanpiere, Mary A Young, Seble Kassaye

**Keywords:** Pre-exposure prophylaxis, HIV, Prevention, Women, United States

## Abstract

**Objectives:**

The latest advancement in HIV prevention, Pre-Exposure Prophylaxis (PrEP), could reduce incidence among women. However, PrEP uptake has remained low among US women since its approval in 2012, while use has increased among men who have sex with men. This study addresses women’s knowledge, attitudes and potential behaviors regarding PrEP. While HIV-negative women are the potential users of antiretroviral (ARV) medications for PrEP, HIV-positive women who have used ARVs could contribute immensely to our understanding of the complexities related to taking such medications. This study is the first to synthesize the opinions of both groups of women.

**Methods:**

We conducted eight focus group discussions, segregated by sero-status; four with at-risk HIV-negative (20) and four with HIV-positive (19) women in Washington DC during 2014. Topics discussed include PrEP awareness, likelihood of use, barriers and target populations.

**Results:**

PrEP awareness was almost non-existent and the HIV-negative women urged publicity. They expressed much enthusiasm about PrEP and wanted to use and recommend it to others despite recognizing potential complexities related to taking PrEP, such as side effects, access, duration and frequency of use. HIV-positive women were less supportive of PrEP for those same reasons based on their experience with taking ARVs. They preferred condoms over PrEP given relative efficacy, affordability, accessibility, and prevention of other STIs.

**Conclusions:**

There is an urgent need for PrEP public health campaigns catered to the needs and concerns of women, most importantly bolster their awareness of PrEP.

## Introduction

In 2013, nearly 20% of new HIV infections in the United States (US) occurred among women [1]. Of these new infections, 86% resulted from heterosexual contact with a high-risk male [2]. Black and Latina women are at increased risk of acquiring HIV compared to all other racial/ethnic groups of women [3]. In 2013, black and Latina women accounted for 63% and 15% of all new HIV infections among women in the US, respectively [4]. The latest advancement in HIV prevention, Pre-Exposure Prophylaxis (PrEP), could potentially contribute to reducing HIV infection rates among women [5]. PrEP is a biomedical method that uses antitretroviral medications (ARVs) to prevent HIV in uninfected individuals who are at high risk of becoming infected [6]. In 2012, the US Food and Drug Administration (FDA) approved oral Truvada (tenofovir disoproxil fumarate and emtricitabine) for PrEP among sexually active adults at risk for HIV infection [7] based on two clinical trials [8,9]. The CDC developed interim guidance for PREP use between 2011 and 2013 for men who have sex with men (MSM) [10], heterosexually active adults [11], and injection drug users (IDU) [12], followed by comprehensive clinical practice guidelines in May 2014 [13].

Since the approval of Truvada for PrEP in the US, its use has increased considerably among men but remained static for women; a nationwide analysis of PrEP uptake using pharmacy databases showed that the absolute number of females who started PrEP in Quarter 1 of 2012 was 159, and remained flat for 11 quarters (over 3 years) until quarter 3 of 2014; for the same time period, the number of male PrEP users rose from 153 to 1064 [14–16]. The investigators suggest that the increase in PrEP prescriptions in men relative to women is related to growing awareness of PrEP among the MSM population; however, it is not clear why awareness and use among women have not similarly increased.

US women’s knowledge, attitudes and opinions of PrEP have not been studied extensively. The few studies available reported very low awareness of PrEP among women; however, once informed, high-risk HIV-negative women in these studies expressed willingness to use PrEP [17–20]. Auerbach et al. [17] conducted 12 focus groups in six cities with 144, mostly black, women. Smith et al. [18] conducted mixed gender focus groups among young African American urban men and women in which 35 women participated. Wingood et al. [20] examined racial differences and correlates of potential PrEP uptake in nationally representative samples of 1509 unmarried African American and white women aged 20–44 years and found that African American women were more likely to report potential use of PrEP. Peer approval was an important facilitator for PrEP uptake among women [17,19,20]. Flash et al. [18] conducted five focus groups in 2012 prior to FDA approval of PrEP with 26 black women comparing women’s opinions on oral versus topical PrEP. Their findings suggest a need for an HIV prevention strategy that women can control. Young and McDaid’s review of the literature on PrEP [21] concluded that more studies are needed to understand acceptability of PrEP amongst at-risk individuals. Further, the scope of the previous studies [17–20] was limited to HIV-negative women. There is another group of women - women with HIV-who have used ARVs and thus could contribute to our understanding of the complexities related to their use, including the side effects, logistics and social factors. Unfortunately, HIV-positive women’s experiences are often neglected in development of HIV prevention programs [22]. This study offers an opportunity to hear HIV-positive women’s insights into the facilitators and barriers associated with taking ARVs and advice based on their experience, which could be useful in designing educational campaigns catered to the needs and concerns of HIV-negative women.

Findings presented in this paper are part of a larger qualitative study on women’s knowledge, attitudes, and potential behaviors (KAB) of biomedical prevention strategies, namely, PrEP and Treatment as Prevention (TasP). This study was conducted among the participants of the Women’s Interagency HIV Study (WIHS) Washington, DC site. The WIHS is the largest prospective, observational study of HIV-infected and at-risk HIV-uninfected women in the U.S [23]. This multicenter ongoing cohort study monitors participants through semi-annual visits, which include interviewer-administered survey instruments, physical examination and specimen collection. This paper focuses on HIV-positive and HIV-negative women’s awareness of, attitudes towards and potential use and/or endorsement of PrEP.

## Methods

We used focus group discussions method for this study as it can provide insights into complicated topics, particularly when the area of concern relates to multifaceted behavior or motivation [24]. Focus groups provide a conducive format to uncover factors that influence women’s opinions, behaviors, and/or motivation to take PrEP or to promote/discourage PrEP use among important others.

### Focus group discussions

We conducted focus groups segregated by HIV sero-status with Washington DC WIHS women during February - May 2014. At the time of this study, there were 309 active participants in the DC WIHS site; of them 218 were HIV-positive and 91 were high risk HIV-negative. A recruitment letter was sent to the participants informing them about the study. An announcement was also placed in the DC WIHS newsletter. A total of 80 women expressed interest in the study and eventually 39 participated in the focus groups; they were scheduled in the order in which their calls were received, and based on their HIV status and availability. In all, eight focus groups were conducted, four with HIV-negative women and four with HIV-positive women; each included between three and eight participants, with an average of five per group. Participants signed the informed consent form approved by the Georgetown University IRB. Each participant received $40 cash, transportation assistance and refreshments. Focus groups were conducted in a private conference room at the Washington DC WIHS study site, which lasted from 1.5–2 hours. The discussions were digitally audiotaped and subsequently transcribed by the study staff.

### Participants

A total of 39 women-20 HIV-negative and 19 HIV-positive-participated in the focus group discussions. Both groups were similar in median age, education level, income level, housing status and employment (Table 1). Their ages ranged from 31 to 62 and the median age was 49. Of the 20 HIV-negative women, 16 were African American, two were Latina, and two identified as “other.” All HIV-positive women were African American.

The majority (65%) of HIV-negative participants reported having at least one male sex partner in the last 6 months and only 25% of them used condoms. All HIV-positive women except for one long-term non-progressor had used ARVs and all but two reported 95- 100% adherence in the last 6 months. At the time of the study, two HIV-positive women were not on medication. HIV-negative women, as participants in the larger WIHS study, are tested for HIV during their WIHS semi-annual visit. They all tested HIV-negative within the prior 6 months. All participants were given pseudonyms to ensure confidentiality.

### Research topics

Key topics discussed in the focus groups and presented in this paper include HIV-negative women’s experiences with HIV; perceptions of their own risk of HIV infection; HIV-negative and HIV-positive women’s awareness of PrEP; acceptability of PrEP; preferred HIV prevention method; concerns about the PrEP package; and potential target groups for PrEP outreach. Focus group guidelines were developed around these topics, and modified based on the discussions. The main topics were discussed in all groups.

After ascertainment of prior knowledge of PrEP, a script based on CDC guidance documents [25] was read to the participants describing what is PrEP, FDA’s approval of PrEP and the different aspects of the comprehensive PrEP package. This was then followed by further discussion to understand women’s thoughts and concerns about PrEP. The lead author moderated all the focus group discussions.

### Data analysis

All the focus group discussions were digitally recorded and later transcribed verbatim by two of the co-authors (initials here). Data were coded and analyzed using NVivo 10 qualitative analysis software. Codes were developed based on the domains of knowledge, attitude and potential behaviors. They were further refined and agreed upon. Two of the co-authors (LSE and NCP) coded the data to facilitate inter-coder reliability and the lead investigator reviewed the coding. The study team iteratively discussed the data: after each focus group, and during and after transcriptions and coding. We listened to the recordings several times and discussed emerging themes and patterns.

## Results

Key findings from this study are as follows: (1) all the HIV-negative women in the study have family members with HIV and perceive themselves to be at high risk of acquiring HIV; (2) awareness and knowledge of PrEP is almost null among all women; (3) women’s reactions to PrEP differed based on their sero-status: HIV-negative women expressed much enthusiasm while the HIV-positive women voiced caution and concerns based on their experience with ARVs; (4) HIV-negative women overwhelmingly wanted to recommend PrEP to others, but HIV-positive women were not keen; (5) HIV-negative women agreed that the combination of PrEP plus condom would be the best preventive approach, while HIV-positive women preferred condoms alone; (6) women have questions and concerns about the PrEP package; and (7) sex workers and sero-discordant couples could be potential target populations but PrEP may not be suitable for young adults. These findings and other concerns raised by the women are detailed and discussed in the following sections.

### HIV-negative women’s experience with HIV and risk perceptions

The HIV-negative women in the study reported having some experience with HIV, even though they did not have HIV themselves. 19 out of the 20 women reported that they had family members or friends who were HIV-positive—one woman’s mother, another woman’s brother, someone’s sister, ex-boyfriend, cousin, friend; sometimes more than one. Some of the women acted as support systems and witnessed health-related problems and suffering associated with HIV infection among close relatives or friends. For example, in one focus group of five women, four had family members with HIV.

JADA (HIV−): My mom was HIV-positive and I was her support system.

BRITTANY (HIV−): It’s my brother.

SABRINA (HIV−): I had two sisters that died from HIV.

ANNA (HIV−): I was engaged to a man that [had] full blown AIDS.

Some talked about how this exposure motivated them to stay HIV-free.

JADA (HIV−): I’ve just seen my mother suffer with different illness, not just with the virus, but the virus attacked her brain thing. […] And—and—and that affect me a lot. ‘Cause when I was really strung out, I stopped taking all that risk behavior!

Most of the HIV-negative women perceived themselves to be at risk of getting HIV almost constantly. Such perceptions were based on their current risk—caused by their partners or themselves, through multiple partner sex or drug use; their past risky behaviors, past or current trauma, and what they see around them in their communities.

JADA (HIV−): When aren’t you in risk? I’m married. And I really don’t think my husband cheated on me…but you have found so many cases [emphasis]. Being married and being in a wholesome relationship, and everything. And bam! Somebody cheated. So I feel like my life was always be at risk.

[Several participants agree with JADA: “yeah”, “yes”]

A 49-year-old woman explained how her concerns have changed over time from “I may get caught [cheating]” to “I may get HIV.” She also exposed another component of risk perception, how people focus on partners’ risky behaviors but not their own.

RASHANA (HIV−): After separating from [my son’s] father, after a few years, I found out that he was a hypodermic drug user. And for about six years, like without fail, I went to my doctor every six months and got tested because I was terrified. It never once occurred to me that—that my own behaviors, could have made me high-risk.

Rashana reported that she is not involved in high-risk behavior anymore and has only one partner whom she trusts yet her previous experience haunts her.

RASHANA (HIV−): I do trust my partner...but there’s that nagging doubt in the back of my head, that says, “Well it’s possible he might be cheating or something.” But I—I know in my heart that he’s not, you know. Um, but would you suggest using condoms even if you have that faith in your other half? I mean, as AIDS at such a high and prevalent risk at- in these days and times now, even with the treatments that you have?

HIV-negative women saw high risk around them and were worried about acquiring HIV even when their circumstances suggested that they were not at immediate risk. They were apprehensive about HIV in their communities.

JULIANA (HIV−): Uh, at this current time, I am not seeing anybody, I am not having sex. And, in my little world, I feel like I’m protected but I know that in the larger world I am not.

In terms of HIV risk, the women talked about husbands who sleep with others, get HIV and pass it on to their unknowing wives; about women who are angry that they got HIV and pass it on to other men; men and women who have not used condoms even when they knew that their partners were HIV-positive; drug parties of yesteryears where multiple partner sex was common; and about the people in their families who have had HIV, their suffering and sometimes death; their boyfriends and husbands who were not honest. Some women talked about being raped. They talked about their partners or themselves using drugs. These are the risky situations that either they were involved in currently or in the past, or have seen among their families and friends; all these factors added to their anxiety that they may acquire HIV. In summary, these are the fault lines in women’s lives that often expose them to HIV.

### Lack of PrEP awareness

Of the 39 participants, only five had heard of PrEP – one seronegative and four seropositive women. The one HIV-negative woman had learned about PrEP in the context of gay men. The women were not aware that PrEP is approved and could work for women too.

LAURA (HIV−): I hear it with the gay community. We use it a lot in gay populations…not with women though.

SAKINA (HIV−): I ain’t never heard of this!

RASHANA (HIV−): And to be honest what the—I guess is it PrEP? Which would be for people like us that aren’t infected. For—at the sake of sounding stupid, as much work as I do in the community [peer recovery specialist], I’ve never heard of it.

SHANA (HIV+): I heard that, um, it was like a few bleeps over the news that there was a possible pill or vaccination that a person who was negative could take to probably help them from contacting the virus.

Women were surprised and angry that they had never heard of PrEP and expressed concerns about lack of awareness and information. They only hear about condoms and abstinence as prevention methods but not PrEP. They strongly recommended that there should be educational campaigns to inform people about PrEP. Some were so upset and angry that that they had never heard of PrEP, declared that lack of awareness was the only barrier preventing people from using PrEP.

LAURA (HIV−): Lack of awareness. at’s the only barrier I’ve seen.

SAKINA (HIV−): But a lot of people need to know about this, you know. Especially people who try not to get it and try to live right and do the right thing and don’t know this thing is out here to help them [shows paper with PrEP information shared with the group], you know. Some people just don’t know about this [knocks on table]. […] And everybody has a right to know what decision they can make to prevent this, to help save the next generation.

JORDAN (HIV+): But when they talk about HIV, they should talk about this [PrEP] as a prevention. […]When you hear prevention about HIV, you hear condoms or no sex. Those are the only two preventions you hear…I don‘t understand why it’s [PrEP] not as advertised as anything else.

Without prompting, the women often advised how PrEP should be advertised all over the city, on the buses, metro stations (subways), on doors everywhere, in doctors’ offices, social media*-*everywhere possible, with phone numbers to contact, particularly on streets and public places because “I learn my information from the street.” Some said it helps to have an incentive attached, while others pointed out that prevention of HIV itself (saving your life) *is* the incentive.

SAKINA (HIV−): They should have that on—hangin’ up so—so people can get, you know, notes. ‘Cause I read everything that I see on doors.

QIANA (HIV−): Me too.

SAKINA (HIV−): Put it on doors and Safeways [grocery store] and stuff.

QIANA (HIV−): On doors [crosstalk], on the bus stop. Put it on the side of the bus.

SAKINA (HIV−): People read that! Subway station. They will read that.

### Different reactions based on HIV status

#### Enthusiasm and hope from seronegative, caution and concern from seropositive

Women’s reactions vastly differed based on their HIV sero-status when they heard about the details of the PrEP package and the potential side effects. PrEP package includes getting a prescription from a provider, using the pill every day consistently and visiting a provider for HIV testing blood work and prescription every three months [13]. Further, the individuals are advised to use condoms along with PrEP. The potential side effects include gastrointestinal symptoms including nausea, fatigue, and damage to the kidneys and bones. HIV-negative women reacted unanimously with much enthusiasm and praised PrEP as lifesaving; HIV-positive women expressed great concern and advised caution. HIV-negative women wanted to know where and how to get PrEP; HIV-positive women asked why medicate otherwise healthy people. HIV-negative women said they would use PrEP along with condoms; HIV-positive women asked why not simply use condoms.

Indeed, HIV-negative women’s immediate reaction was elation to learn about PrEP and they had lofty expectations of its potential impact. They felt that *everyone* would benefit from PrEP, particularly those at high risk and “whoever is having sex.”

JADA (HIV−): I think that was amazing! It’s amazing! Ya know that they have something, now, to try to prevent you from getting the virus.

SAKINA (HIV−): This is a very good thing that probably will save the- most of the world!

Some went as far as to say that every sexually active HIV-negative person should be put on PrEP.

ANNA (HIV−): Well, if you’re not havin’ sex then that’s the only reason to me to not to need it, you know. For real. Because you have too many women out here that have it because of their husbands. And then the husband come to women that’s out there that’s gonna do what you don’t wanna do, you know. Even the man from the church does those things. So if you wanna be blind and trust your man and y’all not talk about this, you know, and take it. is is needed, you know. This is a epidemic. This is killin’ the human race.

The women said they would use PrEP based on their own or their partners’ risky behaviors. Some asked whether the research team could provide them with prescriptions right away.

ANNA (HIV−): well, if that pill comes then I ain’t takin’ any chances. I don’t care who it is. Give it to me. Give it to me.

MEGAN (HIV−): ‘Cause I just-I just hope it succeed though. I just hope it succeed because I wanna be the first one to take it. Because I been doin’ a lot of stuff that I have no business doin’.

Some women did not feel the need to take PrEP either because they are in monogamous relationships or not sexually active. However, they said they would have used PrEP in the past when they had multiple partners or used drugs, or would use it in the future if their relationship status were to change.

QIANA (HIV−): No. I wouldn’t [need PrEP now]. But in my youngin’ and youngin’ days [Sakina laughs], I would need PrEP! I would need PrEP ‘cause I was very promiscuous. I—I mean, I had my fun. But I—I found someone, I’m a happy camper. I have no reason to mess around [a participant laughs]. I don’t—I don’t see nothin’ out there that interests me and I’m scared.

FELICIA (HIV−): If I were a single woman, if somethin’ happened to my husband tomorrow and I had to go back on the dating scene and all that stuff, I would probably—I would still want the, um, I—I would be a proponent to take the PrEP. I would want to take it.

In their initial reactions, the HIV-negative women did not focus on the details of the PrEP package. When specifically asked how they feel about quarterly doctor visits, blood work and side effects, they still brushed away the potential difficulties and remained focused on the availability of PrEP. They saw PrEP as something that can “end the epidemic,” “save the human race,” and save their lives and their children’s. Choosing to take PrEP was seen as an act of responsibility for one’s own health:

CYNTHIA (HIV−): A lot of people don’t take responsibility. But that’s not my prob—I don’t care. That’s on them if they don’t. I’m taking responsibility. I’m looking out for me. […] I’m taking it to keep myself healthy. And I don’t give a fuck of what you think about it!

TAWANA (HIV−): Going to the doctor’s every day, every week—I mean every month- every three months, we do that anyway. Because some of us do it every single day. Um, because that’s how risk—I mean risk takers we are [sex workers community]. Um, so yes, that—that will be something really good for the community.

However, HIV-positive women’s reactions completely contrasted with those of HIV-negative women. They approached PrEP with caution and concerns. Their immediate focus was on the logistical issues related to using PrEP such as getting it prescribed, quarterly doctor visits, blood work, copays, and potential side effects. The women compared the costs, accessibility, and the ability to prevent multiple sexually transmitted infections of condoms against PrEP. They felt that the different components of the package made PrEP use too complex and difficult to manage; using condoms appeared to be the better option. PrEP could bring on additional problems such as side effects. For the HIV-positive women, all these issues seemed to outweigh the benefits of PrEP.

ANGEL (HIV+): Sounds real complicated for-to someone who is HIV negative... at sounds like a eight hour job. I gotta take this pill every day. I gotta go see the doctor to get the pill. And then if I’m private insurance or Medicaid or however I got a copay or I gotta be put on this list of people that take Truvada because maybe I’m into that—I’m in a high-risk population over here of contracting it and then how do I hide this? And that goes back to HIV medicine. Maybe I don’t want my partner to know that I’m takin’ this. Where do I put it? [...] Its like, “Screw that. I’m just gonna put on a condom and go.”

JASMINE (HIV+): Buyin’ a condom at CVS is a heck of a lot cheaper than schedulin’ a doctor appointment, payin’ copay, pickin’ up the prescription, remember getting tested. […] If I have to make a choice between buyin’ a condom and doin’ that I’m gon’ buy a condom.

JORDAN (HIV+): It just seems that [the] package definitely was very discouraging. Like, the more you talked about it, it just, it was, like, it was very discouraging, the package.

In one focus group, the women discussed the side effects among themselves:

ANGEL (HIV+): I’m young. I’m healthy. Kidney?

JASMINE (HIV+): Yeah.

ANGEL: Headache? Nausea? Diarrhea?

JASMINE (HIV+): Mm.

CHELSEA (HIV+): Weight loss.

JASMINE (HIV+): I’m not takin’ that, you know. That’s not doable, you know. Yeah, I’mma catch this even though they put the side effects on TV. I think that’s so cute. For-to protect myself from contractin’ HIV but then takin’ this pill every day, it’s subsequently that I’mma have all these other problems that I don’t have already, you know.

They saw PrEP as medicating otherwise healthy people; that it intervenes with women’s bodies, and more importantly, that PrEP is limited in its effectiveness as it can only prevent HIV.

ALISSON (HIV+): I don’t feel like they should put a medication out that’s going to cause, you know, another medical problem.

ANGEL (HIV+): ... at’s prevention from contractin’, you know, that particular chronic disease [HIV] but there’s those other STDs and STIs out there… Use two condoms, I don’t care!

We asked the women what would be their preferred HIV prevention method: condom alone, PrEP alone or PrEP plus condom. HIV-negative and -positive women differed again in their opinions. The HIV-negative women reported that PrEP plus condom would be their preferred method. They explained that PrEP is a “semi-safety net,” a “big backup,” an “extra protection tool,” in the context of condom breakage, and that it gives them control on saving themselves. They explained that PrEP was a necessary additional tool for protection because many of them had experienced condom breakage. In contrast, HIV-positive women pointed out that PrEP only prevents HIV but no other STIs or pregnancy; whereas, condoms prevent HIV, STI and pregnancy and do not interfere with women’s bodies. They preferred that their daughters depend on condoms rather than using PrEP. In every respect, HIV-positive women immediately saw problems in PrEP use. They compared PrEP to condoms and determined that condoms were still the better option.

A few HIV-negative women felt that PrEP would decrease condom use and as a result, STDs will be on the rise.

MADISON (HIV−): It’s (STDs) gon’ be on the rise. Because they are not gonna use no condoms. ‘Cause all they worry about is not gettin’ HIV.

Still others thought that PrEP might, in fact, increase condom use as men (partners of HIV+ women) weigh between the side effects of PrEP versus inconvenience of condom use.

JORDAN (HIV−): Mhm! Definitely. Uh-huh. Mhm. He would definitely be more open. Right, he would. He would, yeah. He would probably--he would probably use condoms more often.

MICHELLE (HIV−): ‘Cause the condoms don’t have many side effects. It’s not gon’ make him sick… It’s not going to do anything to his kidneys or anything like that. So, I think people will probably be thinkin’ about that, “I can put on a condom and my kidneys will be okay.” Or, “I don’t have to worry about getting sick.”

However, the HIV-positive women were less optimistic that PrEP would change sexual behaviors.

BARBARA (HIV+): No. I think not, um, if so many people are being diagnosed HIV didn’t change it, what makes you think that PrEP is going to change it?

### Recommending PrEP to Others: “Yes! Yes! Yes!” vs. “I’m not gonna push it”

We asked the women whether they would recommend PrEP to other women close to them, such as their daughters and nieces. Again, the HIV-negative women responded with a resounding “yes,” while the HIV-positive women limited their role to “informing” but “not recommending.”

The HIV-negative women were just as enthusiastic about recommending PrEP as they were about taking it themselves.

MODERATOR: Would you tell your daughters, your sisters, your…

BRITTANY (HIV−): Yes!

CYNTHIA (HIV−): Yes.

MODERATOR: …nieces. Your female—other relatives?

CYNTHIA (HIV−): Yes.

BRITTANY (HIV−): Yes! Yes! Yes! Yes! Yes! Mhm. Yes [crosstalk with moderator]! Not only for um females [chuckles]. Nephews. Uncles. I would…

JADA & CYNTHIA (HIV−): Yeah! Yeah!

CYNTHIA (HIV−): I would.

BRITTANY (HIV−): …depending on the lifestyle. You know so—I mean—I would recommend it.

CYNTHIA (HIV−): Yeah. To save their life [crosstalk]!

BRITTANY: Strongly, strongly, strongly.

CYNTHIA (HIV−): Yes, I would do.

JADA (HIV−): Oh, yeah! I’m a be talkin’ about it for weeks.

BRITTANY (HIV−): Definitely! Definitely.

Some women wanted the research team to organize a PrEP information sessions for their daughters.

The HIV-positive women said that they would inform their female relatives and friends about PrEP but would not necessarily recommend its use. They want them to make informed decisions but their recommendation would be to use condoms. They saw PrEP as medicating otherwise healthy bodies.

JASMINE (HIV+): I would tell them about it. I don’t know necessarily if I would, per se, put a gun to their head and say, “Yeah, you better take this.”… Condom, yes.

ANGEL (HIV+): I’d say, “Look. I’mma give you some condoms. You put ‘em on. […] I will educate them on it (PrEP). I’m not gonna push it. I’m more for condoms, female and male. […] I’m more for condoms than Truvada because of the side effects.

However, the HIV-positive women had mixed opinions about recommending PrEP to their sero-negative partners.

ALEXANDRA (HIV+): I wouldn’t recommend it to him. If I’m still undetectable, I’m taking my meds, everything is going fine, why would I […] and the chances of me transmitting to him are so low.

Tiana at first did not want her partner to use PrEP because her viral load is undetectable and she uses condoms, but changed her mind after putting more thought into it.

TIANA (HIV+): I would recommend it. If it’s gonna protect from gettin’ HIV; but also with the pill, I would suggest condoms.

Another woman explained that her HIV-uninfected husband was not willing to use condoms and that it bothered her; if PrEP was available then, she would have recommended it to him, she said. However, her current partner uses condoms.

Gabrielle (HIV+): I probably would have definitely recommended it to my husband, ‘cause he didn’t want to wear the condom. But my friend, he wears the condom. And I would recommend it to him now, but I still would probably use the condom or that would be like an option.

Married women or those in committed relationships were more willing to take on the responsibility themselves to keep their viral loads low with medication in order to protect their partners from HIV and the side effects of PrEP.

They saw PrEP as an option for HIV-negative male partners who are unwilling to use condoms.

### Questions and concerns about the PrEP package

Upon having a heightened awareness of PrEP and its important role in HIV prevention, many participants expressed favorable attitudes regarding PrEP. However, both HIV-negative and HIV-positive participants also expressed apprehension about the complexities related to using PrEP, particularly potential side effects, access, duration of use, and possible resistance to Truvada.

The potential serious adverse effects of Truvada, namely decreased kidney health and loss in bone density, were the first and most important concern of HIV-negative women. Participants agreed that more research on Truvada’s possible long-term side effects would help them in their decision to take PrEP and/or to recommend it to others.

CYNTHIA (HIV−): I would still want to know the long terms of it. What, ya know, if taking anything for a period of time does something to your body. That’s just to me common sense.

However, for those who felt most at risk, the benefits of using PrEP outweighed the side effects.

JADA (HIV−): I’m worried [about side effects]—(but) I’m concerned about saving myself from high-risk livelihood (sex work).

SAKINA (HIV−): It ain’t only just PrEP. Any medication has side effects.

CYNTHIA (HIV−): Anything you take is gonna have side effects. So, it just depends on whether you’re willing to take this pill every day, to save your life.

The common but short-term side effects including headaches, weight loss and gastrointestinal problems, like nausea, diarrhea or abdominal pain, did not concern the participants. Almost all had experience with taking daily medications and such side effects.

In contrast, the HIV-positive women expressed stronger concerns about side effects of HIV medicines and suggested that they would be too burdensome for widespread PrEP use. All the HIV-positive women in the study, except for one long-term non-progressor, have used HIV medicines and several have experienced side effects.

JASMINE (HIV+): My kidneys went into failure using Truvada. And I was on dialysis for a year. Almost took my life. […] I would not want them to deal with, you know, with what I deal with. But I would also let them know my concern for side effects because if somethin’ were to happen to them and their side effects. Like that’s gonna bother me either way. […] It may not happen that way for a family member or, um, somebody that I love, but I had had so much going on, it’s just, that thing terrorized me! I’m serious...life-altering decisions, or life-changing things. It’s not a good thing.

JORDAN (HIV+): [PrEP is] just a lot, like the long-term, it’s just so many questions about the long-term effects with the package. It’s just – it’s just like, do you just kind of chance it and weigh it out and see, or do you deal with these known side effects?

MADISON (HIV+): And then what is that pill gon’ do to the body? See you got that body, and I lost a beautiful figure due to the HIV.

A participant tried to picture herself taking PrEP back when she was HIV-negative and said she would have immediately stopped because of the side effects.

ANGEL (HIV+): [If] they had Truvada back then and [with] them side effects. If I had started havin’ diarrhea and nausea, yeah. I woulda tried it. But I woulda stopped…if I’m constantly throwin’ up or constantly gotta stay by the bathroom, that’s not gonna happen. I’m gonna go back to condoms and I’mma tell my doctor, “ ank you but no thank you.”

The same participant, when we told her group that the HIV-negative women were enthusiastic to use PrEP, immediately asked whether we had told them about potential side effects.

ANGEL (HIV+): Did y’all tell them side effects of that?

While most HIV-negative women were not concerned with having to go see the doctor regularly to get their PrEP prescription renewed, a few thought that condoms were comparatively much more accessible.

RASHANA (HIV−): Let me ask you a question. I could go into the local health clinic and get condoms free. Can they walk in and get this drug free?

HIV-negative women wanted to know for how long they need to use PrEP and were disappointed to learn that they have to use it as long as they are at risk of getting HIV.

JADA (HIV−): For how long? Forever in life?

MODERATOR: Yeah, uh, yes.

PARTICIPANTS (several): Oh. [Disappointed]

MODERATOR: As long as you’re, ya know, at risk for acquiring HIV.

JADA (HIV−): Oh. Yeah. Oh, that’s forever in life! Psych! [laughs]

Opinions about taking PrEP daily were mixed. Several HIV-negative women were concerned about having to take it every day.

JULIANA (HIV−): You know what would be good is, um, my sister takes a bone density pill that she takes once a year. There’s the morning after pill. There’s a whole bunch of things like that. What would be good is if you didn’t have to take this one every day of your life. [...] It would be good if there were a pill that you could take once a year.

Others thought that daily use would make it easier to remember and remain adherent to PrEP.

KEISHA (HIV−): But how is it administered?

MODERATOR: Once a day.

KEISHA (HIV−): Yeah one—and that makes it easy.

SAKINA (HIV−): Yeah. Very easy.

Some saw PrEP as such a necessity that they would simply not forget to take it.

MEGAN (HIV−): I think for a pill like that I don’t think you—you can’t forget to take that.

ANNA (HIV−): You don’t forget that.

MEGAN (HIV−): Yes. As soon as you wake up [laughs].

TAWANA (HIV−): It’s—it’s like something like a vitamin a day. Because I know that I need that vitamin, so it’s going to be taken.

JADA (HIV−): No. No. No. You got to. You got to [take].

Furthermore, the majority of the participants had experience with taking medications every day and thus saw PrEP as just another pill in their medicine cabinet.

SAKINA (HIV−): I take medication everyday so, I know.

QIANA (HIV−): I take every [crosstalk]—me too.

SAKINA (HIV−): Yes. Yeah.

KEISHA (HIV−): Yeah.

QIANA (HIV−): I’ll just add it to my collection [laughs].

They were also concerned about what happens if they get infected with HIV while taking PrEP.

VANESSA (HIV−): But what if you take PrEP and then you still get it?

The HIV-positive women were concerned whether taking PrEP would make one resistant to Truvada if ever the user gets infected with HIV.

BARBARA (HIV+): Do I want to be on this medication for the rest of my life knowing that I still can contract HIV while I’m on it and then become resistant to the medication?

### Potential target populations: Sex workers and serodiscordant couples

All women, despite their sero-status, felt that PrEP is a viable option for HIV prevention; sex workers were often identified as potential candidates for PrEP.

MICHELLE (HIV+): Are you serious? So let me just understand. I’m a little slow at times. So I am a sex worker [hypothetically].

MODERATOR: Yes.

MICHELLE (HIV+): Negative.

MODERATOR: Yes.

MICHELLE (HIV+): And there are days when I’m unprotected. If I take Truvada every day while I’m living that lifestyle, there’s a chance that I would not contract the virus?

MODERATOR: It would decrease the chance of you getting the virus.

MICHELLE (HIV+): Shut up. I mean, don’t shut up [everyone laughs]. […]I think that is wonderful! I think that is great.

Two participants, who reported to be involved in sex work, thought that PrEP would be helpful to sex workers.

TAWANA (HIV−): Um, because like the- people that I hang with them- we’re all over the world. Um so, to understand that we have a semi-safety net…I just know in my group [sex worker group] that is something that is ACTUALLY needed. Because we’re out here! And we need something, like she said, as a backup

MEGAN (HIV−): Yeah, I’d take it. I’d take ‘em.

MODERATOR: OK, why would you take them?

MEGAN (HIV−): I have a lot of male partners. So, I would take ‘em. I would. I wish they would came out sooner but I’ll take ‘em.

Some saw PrEP as an option for serodiscordant couples as well.

DESTINY (HIV+): I take, you know, the HIV meds. But this is something that, okay, if we do have unprotected sex, at least it’s a little prevention here. A big part of prevention here to prevent you from becoming infected.

Young adults were perceived to be at risk of getting HIV; however, women with jobs and middle class incomes declared that PrEP is not for young people, because “ ey’re irresponsible. And they’re partyin’. They druggin’ and they drinkin’.” PrEP would require “too much commitment” from them.

MEREDITH (HIV−): Yeah, that’s just like them takin’ birth control pills. We have birth control pills, kids still gettin’ pregnant. So you tell them they gon’ have to take this pill every day. Do you really think they gonna remember every day to take their pill if they can’t stop from gettin’ pregnant? Really [laughs]? I don’t think so.

JASMINE (HIV+): You’re asking a younger person to change their lifestyle for a preventative measure.

Some pointed out young adults can get condoms for free or buy them easily whereas PrEP would involve medical insurance and parents.

RASHANA (HIV−): most teenagers have insurance through their parents. Their—they don’t want their parents to know about this.

A few women were afraid that PrEP would give a false sense of security, and thus were not willing to recommend it to young people.

GISELLE (HIV−): I would think that if my daughter wanted to use the drug, I would be fearful that the drug would give her a false sense of reality that then she can continue to engage in risky behavior.

JULIANA (HIV−): For my granddaughter, who is twenty years old, I would prefer to promote the condom and birth control.

Interestingly, false security was mentioned as only one of the reasons; difficulties in accessing PrEP and the commitment needed to continue the regimen remained the primary issues. Some women were concerned about people’s ability to handle the responsibility involved in using PrEP, regardless of age.

JULIANA (HIV−): I don’t think it’s age group. I think it’s more of a person who’s responsible for one. More of a person who really feels that they’re at risk and there’s nothin’ else they can do.

### Use PrEP “to save your life”

The HIV-negative women time and time again expressed concern about “saving” their lives. PrEP appeared to them as the magic pill that would save them and the world from HIV. We asked the women why do they want to use PrEP, why do they want to recommend it to others, why such enthusiasm. “To save your life” was the unison response.

MODERATOR: Okay, so why do you want to use this (PrEP)? Why do you want to—why would you recommend it to people?

CYNTHIA and JADA (HIV−): To save your life.

CYNTHIA (HIV−): To save your life. Period.

JADA (HIV−): To save your life.

BRITTANY (HIV−): Yeah and…and like, condoms do break.

ANNA (HIV−): We’re talkin’ about one pill that would save-could possibly save your life.

SAKINA (HIV−): Anything to save my life and—and—and help my daughters.

QIANA (HIV−): On the flyers you should have a lit— have a heading that said— that says, “Help save yourself.”

## Discussion and Conclusion

This study is the first to examine and compare HIV-negative and HIV-positive women’s knowledge, attitudes and behavior regarding PrEP. Their opinions regarding PrEP use differed vastly and were often in contrast. HIV-negative women expressed great enthusiasm about PrEP and overwhelmingly wanted to use and recommend it to others despite recognizing potential complexities related to taking PrEP, such as side effects, access, duration and frequency of use. For them, risks involved in taking PrEP are tolerable compared to getting HIV-infected. HIV-positive women were less supportive of PrEP for those same reasons based on their experience with taking ARVs. They preferred condoms over PrEP given relative efficacy, affordability, accessibility, and prevention of other STIs. HIV-negative women’s enthusiastic reaction was observed in other studies with US women on PrEP [17–20], particularly among African American women [18]. In contrast, HIV-positive women were less supportive of PrEP based on their experiences with the side-effects and stigma associated with taking HIV medications, as well as the complexity of the comprehensive PrEP package—including quarterly medical visits, routine HIV testing and blood work, and continued condom use. They thought PrEP requires too much commitment from the HIV-negative women. They preferred recommending condoms over PrEP given their efficacy, affordability, accessibility without prescription, and ability to prevent other STIs. Nonetheless, both HIV-positive and -negative participants agreed that PrEP should be made available as an option to women despite their differences in enthusiasm for it.

As discussions ensued and each element of the package was discussed in-depth, the HIV-negative women also voiced concerns, which, nonetheless, did not diminish their enthusiasm. In fact, participants continuously tried to come up with ways to overcome their concerns about daily and long-term use, adverse side effects, regular doctor visits and continued condom use. These barriers to PrEP use were among those identified by other studies on US women and PrEP [17–19].

Our findings suggest that HIV-negative women’s enthusiasm for PrEP stems from anxiety based on the presence of HIV in their families and communities, their experience with condom breakage and near misses of getting infected. PrEP would provide a preventive method that they could control and help “save their lives.”

Even though the participants of this study live in the nation’s capital that has high HIV prevalence [26], their knowledge of PrEP was little to non-existent. At the beginning of the study, we were concerned that the WIHS participants may know more about PrEP compared to the general population due to their participation in a longitudinal cohort HIV study. However, that was proven to be wrong. The HIV-negative women, some of whom are engaged in HIV and peer counseling activities, were very upset and angry that they were not aware of PrEP and strongly recommended educational campaigns. Other studies support this finding [17]. Despite the approval of PrEP for women since 2012, results from Auerbach’s study conducted in multiple cities and our study reveal no change in women’s awareness [17]. Therefore, there seems to be a dearth in knowledge about PrEP among women across the country and an urgent need for dissemination of PrEP information.

PrEP use has shown promise in reducing HIV infection in clinical trials involving MSM and transgender women [9], heterosexual men and women [27] serodiscordant couples [8], and intravenous drug users [28]. However, PrEP trials in heterosexual women, VOICE and FEM-PrEP, did not demonstrate efficacy [29,30]. Blood samples collected from the female participants revealed low adherence to study drugs. A qualitative study following the VOICE trial found that the unknown efficacy of PrEP, and the challenges related to daily use were among the important contributing factors to the widespread nonuse of PrEP among female participants [31]. Our study participants reverberated many of these same concerns as potential barriers to PrEP use.

Further, research has revealed that Truvada does not reach as high levels in vaginal and cervical tissues compared to rectal tissue [32], helping to explain why PrEP was unable to protect women as well as it did in MSM. Women who take PrEP must take it every day in order to achieve high levels of protection [33]. HIV-negative women, who are otherwise healthy, may have difficulty adhering to daily PrEP. HIV-negative individuals who take PrEP have to meet with a medical provider four times a year, while the standard of care for HIV-positive individuals with suppressed viral load and stable immunologic status is twice a year [34]. Thus, the burden associated with PrEP use may be greater than that associated with HIV treatment. This discrepancy may further discourage HIV-negative individuals, particularly those in sero-discordant relationships, from taking PrEP.

The participants were able to explain proper use of condom, yet reported that many of them had experienced condom breakage. Randomized clinical trials have previously demonstrated that breakage rate for male condoms is only about 2.5%, and slippage rate is about 1.1% [35]. Education in proper use of condoms and disadvantages of using double condoms is needed. The HIV-negative women in the study professed that they would use PrEP along with condoms, yet only 25% of them (among the sexually active) have reported using condoms in the last 6 months. The HIV-positive women preferred condoms alone over PrEP. Both these assertions could be impractical in real world settings. PrEP use and condom use need to be promoted simultaneously, particularly in the context of high rates of sexually transmitted infections in the US [36].

PrEP is an important prevention method as it gives control to women unlike condoms; people who are continuously at risk, such as sex workers and individuals in serodiscordant relationships, would potentially be the best candidates for PrEP. The enthusiasm of HIV-negative women demonstrated in this and other studies could diminish once the complexity of the PrEP package hits home. In our focus groups, HIV-negative women did point out that PrEP is asking for “too much commitment,” particularly for young adults. It may be difficult to convince young adults to follow the PrEP regimen. Mature age and experience may make women more responsible for their own health and thus more committed to follow the regimen strictly. Our study participants were relatively older (median age: 50 years), thus our findings may not be generalizable to younger women. Further, most of our study participants were African American women living in the DC metropolitan area; more studies are needed to generalize our findings to other ethnic groups and geographical areas. The concerns raised by the HIV-positive women, in addition to those of HIV-negative women, are important and have implications for PrEP uptake. They need to be addressed by PrEP campaigns. Most importantly, as our participants pointed out, lack of awareness and information about PrEP seems to be a major barrier for uptake at this time; it is imperative to bolster PrEP awareness.

## Figures and Tables

**Table 1:** Demographic characteristics of participants.

Variable	HIV− (N=20)	HIV+ (N=19)
*Race*		
Black/African-American	16 (80%)	19 (100%)
Latina/Hispanic	2 (10%)	0 (0%)
Other	2 (10%)	0 (0%)

*Age*		
Median	50.4	50
Average	48.8	49.8

*Education*		
No schooling	1 (5%)	0 (0%)
Grade 7–11	4 (20%)	3 (15.8%)
Complete high school	5 (25%)	8 (42.1%)
Some college	5 (25%)	4 (21.1%)
Complete college	1 (5%)	3 (15.8%)
Graduate school	4 (20%)	1 (5.3%)

*Employment status*		
Unemployed	11 (55%)	13 (68.4%)
Employed	9 (45%)	6 (31.6)

*Average Household Income per Year*		
$6000 or less	2 (10%)	4 (21.1%)
$6000–$12000	7 (35%)	10 (52.6)
$12001–$18000	2 (10%)	0 (0%)
$18001–$24000	2 (10%)	0 (0%)
$24001–$30000	0 (0%)	2 (10.5%)
$30001–$36000	0 (0%)	0 (0%)
$36001–$75000	6 (30%)	2 (10.5%)
>$75000	1 (5%)	1 (5.3%)

*Housing Status*		
Own house/apartment	16 (80%)	14 (73.7%)
Someone else house/apart	3 (15%)	4 (21.1%)
Parents house	1 (5%)	1 (5.3%)

*Relationship Status*		
Married	7 (35%)	4 (21.1%)
Not married	2 (10%)	3 (15.8%)
Never married	6 (30%)	5 (26.3%)
Divorced	3 (15%)	4 (21.1%)
Widowed	0 (0%)	1 (5.3%)
Other	2 (10%)	2 (10.5%)

*Number of Male Sex Partners*		
0	7 (35%)	3 (15.8%)
1	10 (50%)	14 (73.7%)
2	2 (10%)	2 (10.5%)
3	1 (5%)	0 (0%)

*Current Use of Male Condoms*		
Yes	5 (25%)	13 (68.4%)
No	15 (75%)	6 (31.6%)

*Health Care Provider*		
Yes	18 (90%)	18 (94.7%)
No	2 (10%)	1 (5.3%)
